# Neuromuscular ultrasound in delayed sciatic neuropathy following hamstring tear

**DOI:** 10.1002/pmrj.13294

**Published:** 2024-12-05

**Authors:** Jasmina Solankee, Srivats Srinivasan, Russell Payne, Haibi Cai

**Affiliations:** ^1^ Department of Physical Medicine and Rehabilitation University of Texas Southwestern Medical Center Dallas Texas USA; ^2^ Medical School University of Texas Southwestern Medical Center Dallas Texas USA; ^3^ Department of Neurosurgery University of Texas Southwestern Medical Center Dallas Texas USA

A 45‐year‐old healthy man sustained a left‐sided grade III (complete) proximal hamstring tear from a waterskiing accident. Management of his injury at the time was nonoperative. The patient underwent rehabilitation for his injury and progressed well with physical therapy to the point where he had regained close to baseline strength and mobility. Ten months later, he began to develop weakness in the left leg in addition to sensory abnormalities. On physical examination of the left leg, his strength on manual muscle testing was 4/5 for knee flexion, 4/5 for ankle dorsiflexion, 4/5 for toe flexion, and 1/5 for hip extension. His sensory exam revealed diminished sensation to light touch from the left ankle down. His reflexes were normal. The patient provided written consent for this case report.

Nerve conduction study and electromyography (NCS/EMG) were obtained to further evaluate the patient's clinical symptoms. NCS demonstrated decreased left sural sensory amplitude when compared to the contralateral side. The left tibial and peroneal motor NCS were normal. H‐Reflex study of the left tibial nerve showed no response. On EMG, there was evidence of fibrillations and positive sharp waves in the left anterior tibialis, gastrocnemius, and biceps femoris muscles in addition to reduced recruitment. These findings were consistent with a diagnosis of a left sciatic neuropathy with active denervation.

Neuromuscular ultrasound evaluation of the patient's left sciatic nerve was conducted. Ultrasound showed that the left sciatic nerve was enlarged for an 18 cm course from ischial tuberosity to the posterior thigh (Figure 1). Throughout this course, the sciatic nerve was tethered tightly to the retracted hamstring by scar tissue. Distally the sciatic nerve regained a normal appearance. Evaluation of the patient's right sciatic nerve was conducted for comparison.

**FIGURE 1:**
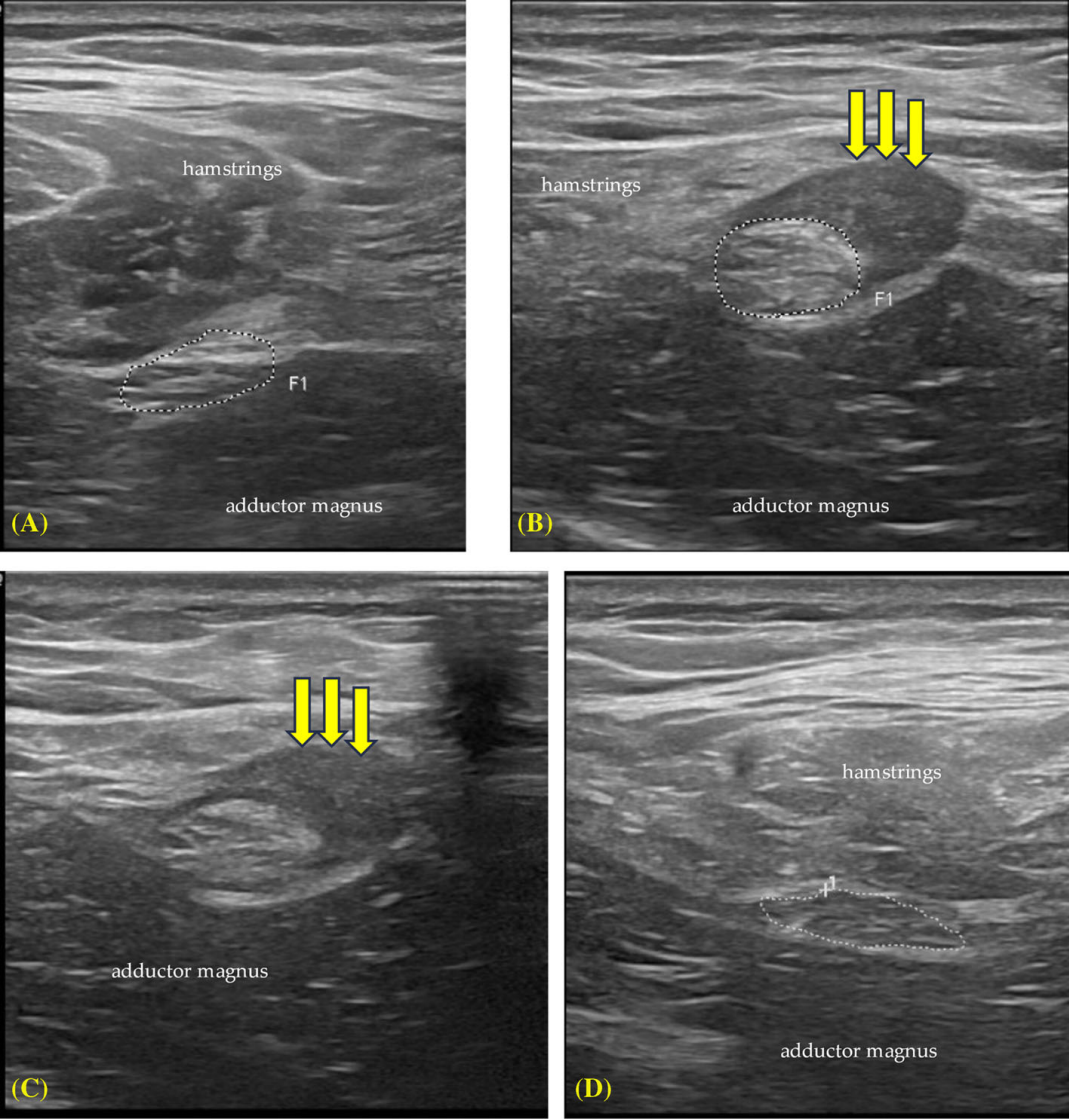
Ultrasound images. (A) Asymptomatic right sciatic nerve in the posterior thigh 9 cm distal to the ischial tuberosity (IT). (B) Left sciatic nerve in the posterior thigh 9 cm distal to the IT. Nerve is enlarged. Hamstrings are retracted with hyperechogenicity and loss of muscle echotexture. Hypoechoic structure (yellow arrows) consistent with developing scar tissue tightly tethers sciatic nerve to retracted hamstrings. (C) Left sciatic nerve in the posterior thigh 16 cm distal to the IT. Again, nerve is enlarged with tethering to retracted hamstrings. Nerve was found to be tethered to hamstrings from IT to 18 cm distally. (D) Left sciatic nerve in the posterior thigh 20 cm distal to IT. Normal appearance.

The patient underwent a left sciatic neurolysis and hamstring tendon repair with neurosurgery and orthopedic surgery. Intraoperatively the sciatic nerve was found to be hypertrophic and tightly tethered to the retracted hamstring for approximately the same 18 cm length demonstrated on ultrasound (Figure 2). At his postoperative follow‐up appointment, the patient reported normal strength and significant resolution of paresthesias and significantly decreased use of neuropathic pain medications.

**FIGURE 2:**
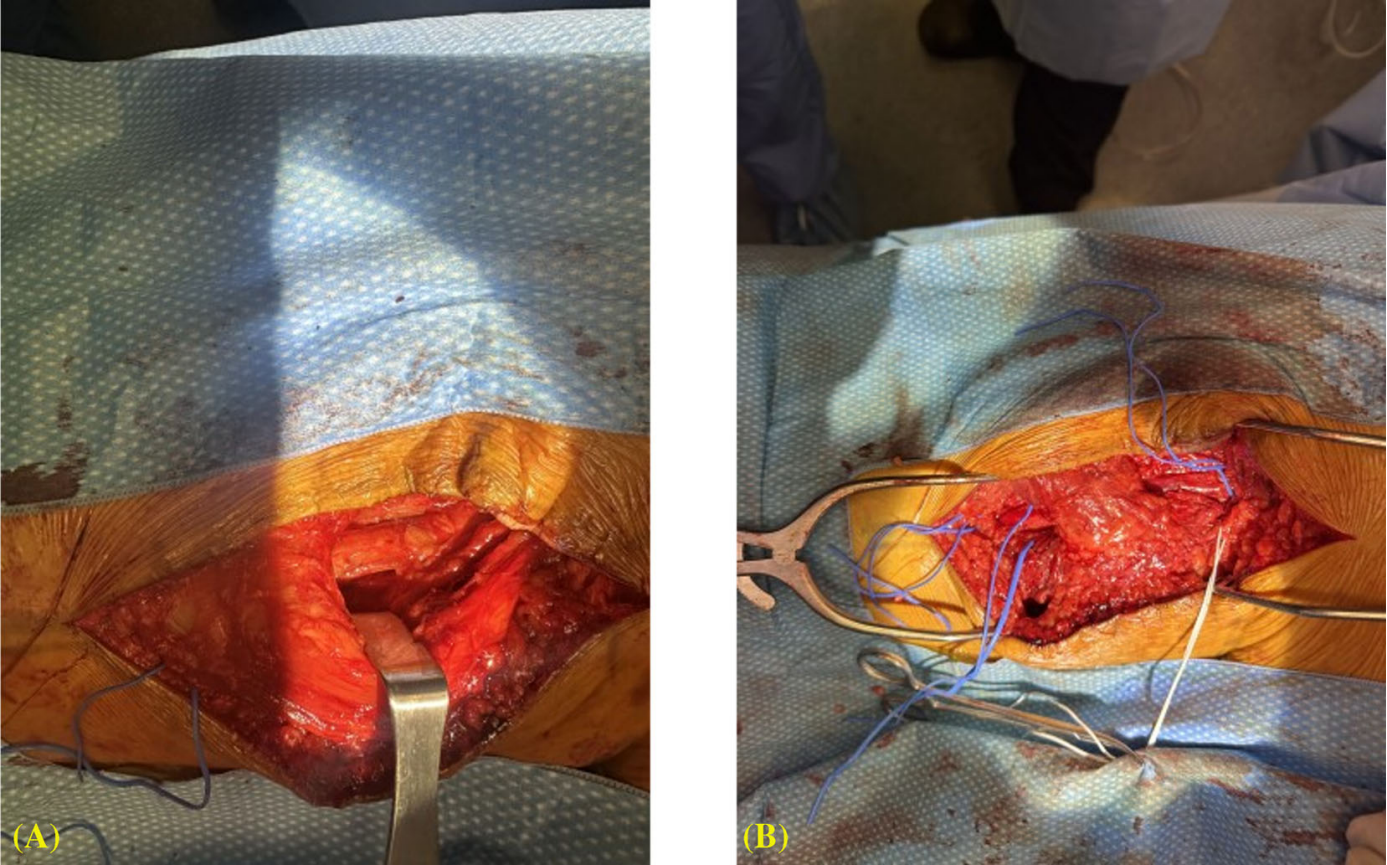
Intraoperative photographs. (A) Thickened left sciatic nerve (blue loop) adhered tightly to hamstring muscle. (B) Left sciatic nerve (blue loops) after partial dissection from hamstring muscle. Posterior femoral cutaneous nerve (white loop).

Nerve injuries can occur because of tendon rupture due to the disruption of the tendon from its normal location. The natural healing process involves creating new adhesions to nearby structures to allow the muscle to regain its ability to contract. Scar tissue adhering to a nerve can result in injury to the nerve and presents with sensory and motor abnormalities. Delayed sciatic nerve injuries in the setting of hamstring injuries, such as seen in this case, have been reported but are underrecognized overall.^1^ Other locations of associated neuropathies after tendon injuries have been documented in the shoulder, elbow, and hand. There have been documented cases of musculocutaneous nerve compression after biceps tendon rupture, median nerve compression after flexor digitorum profundus tendon rupture, and suprascapular nerve compression with rotator cuff tears.^2^, ^3^, ^4^ The utilization of NCS/EMG to determine the presence and extent of these compression neuropathies is well established in guiding surgical intervention to alleviate the compression. In our case, the NCS/EMG was able to identify the presence of a sciatic neuropathy. However, this case highlights the utility of neuromuscular ultrasound in such situations. Ultrasound evaluation assisted in confirming the diagnosis of sciatic neuropathy due to presence of scar tissue tethering the nerve to retracted hamstring muscle. It also allowed for delineation of the extent and length of nerve involvement and how far down the nerve was tethered to the hamstrings. Data obtained from ultrasound evaluation were able to directly guide surgical decision making and planning. This study highlights the importance of adding neuromuscular ultrasound evaluation as a routine part of the diagnostic workup and management of neuropathies resulting from seemingly separate musculoskeletal and orthopedic injuries.

## DISCLOSURE

The authors have nothing to disclose. No funding was received for this study.
